# Synthesis and techno-economic assessment of microbial-based processes for terpenes production

**DOI:** 10.1186/s13068-018-1285-7

**Published:** 2018-10-27

**Authors:** Wenzhao Wu, Christos T. Maravelias

**Affiliations:** Dept. of Chemical and Biological Engineering and DOE Great Lakes Bioenergy Research Center, University of Wisconsin-Madison, 1415 Engineering Drive, Madison, WI 53706 USA

**Keywords:** Isoprenoid, Terpenoid, Limonene, Process systems engineering, Process simulation, Microbial production, Vapor liquid equilibrium, Biphasic fermentation, Fatty acids

## Abstract

**Background:**

Recent advances in metabolic engineering enable the production of chemicals from sugars through microbial bio-conversion. Terpenes have attracted substantial attention due to their relatively high prices and wide applications in different industries. To this end, we synthesize and assess processes for microbial production of terpenes.

**Results:**

To explain a counterintuitive experimental phenomenon where terpenes such as limonene (normal boiling point 176 °C) are often found to be 100% present in the vapor phase after bio-conversion (operating at only ~ 30 °C), we first analyze the vapor–liquid equilibrium for systems containing terpenes. Then, we propose alternative production configurations, which are further studied, using limonene as an example, in several case studies. Next, we perform economic assessment of the alternative processes and identify the major cost components. Finally, we extend the assessment to account for different process parameters, terpene products, ways to address terpene toxicity (microbial engineering vs. solvent use), and cellulosic biomass as a feedstock. We identify the key cost drivers to be (1) feed glucose concentration (wt%), (2) product yield (% of maximum theoretical yield) and (3) VVM (Volume of air per Volume of broth liquid per Minute, i.e., aeration rate in min^−1^). The production of limonene, based on current experimental data, is found to be economically infeasible (production cost ~ 465 $/kg vs. market selling price ~ 7 $/kg), but higher glucose concentration and yield can lower the cost. Among 12 terpenes studied, limonene appears to be the most reasonable short-term target because of its large market size (~ 160 million $/year in the US) and the relatively easier to achieve break-even yield (~ 30%, assuming a 14 wt% feed glucose concentration and 0.1 min^−1^ VVM).

**Conclusions:**

The methods proposed in this work are applicable to a range of terpenes as well as other extracellular insoluble chemicals with density lower than that of water, such as fatty acids. The results provide guidance for future research in metabolic engineering toward terpenes production in terms of setting targets for key design parameters.

**Electronic supplementary material:**

The online version of this article (10.1186/s13068-018-1285-7) contains supplementary material, which is available to authorized users.

## Background

Recent advances in metabolic engineering enable the use of microbes such as *E. coli* and *S. cerevisiae* for the production of chemicals [1–12]. Compared to traditional fossil fuel-based processes, bio-processes can be advantageous for their mild production conditions and good selectivity toward a specific product [13]. Also, the chemicals can be produced directly using microbes instead of being converted via multiple conversion steps (some of which can have low yield and high cost) from fossil fuel feedstocks.

Terpenes (also known as isoprenoids or terpenoids) are a class of organic compounds biosynthetically derived from isoprene (C_5_H_8_: CH_2_=C(CH_3_)–CH=CH_2_) and can be classified into groups according to the number of carbons they contain: hemiterpene (C_5_, i.e., isoprene), monoterpenes (C_10_; major interest of many studies), sesquiterpenes (C_15_), diterpenes (C_20_), triterpenes (C_30_), etc. [14–17]. Terpenes have been attracting substantial attention due to their relatively high prices and wide applications in chemical, food, cosmetics, pharmaceutical, fragrance, flavor and biotechnology industries [14, 18–23]. Some terpenes, such as limonene and linalool, are also potential drop-in biofuels and platform chemicals to produce other value-added products [24–26].

The microbial conversion of terpenes from sugar via microbes has been reviewed extensively [14, 27–36]. Terpenes can be produced mainly via the mevalonate pathway, or 1-deoxy-d-xylulose-5-phosphate (DXP) pathway. Most terpenes are produced extracellularly, and they are insoluble and lighter (in terms of density) than water, thus forming a top oil phase in the liquid fermentation broth. Note that we do not consider the special case where terpenes fail to form a separate phase from water due to the presence of surface active impurities in the broth. Recent studies indicate that the fermenter can be tuned to favor globule coalescence and thus the formation of a separate phase [37–39]. Also, microbial production of terpenes has demonstrated high selectivity toward a specific product [40]. Although intracellular components (such as amino acids and nucleoids) will be released after cell death, compounds that are insoluble and lighter than water (as the terpene product is), mainly lipids, are actually bound to the cell membrane debris and settle to the bottom, and are thus naturally separated from the product (on the top). Therefore, downstream separation cost tends to be relatively low. In addition, several terpenes (such as limonene and pinene) were estimated to have the potential to reach a promising profit margin in a recent study that identifies economically promising bio-based chemicals [41].

The major properties as well as the market price and volume data for selected terpenes are presented in Table 1. It can be seen that limonene has a relatively large market size and price. Limonene is commonly used as a flavor, insecticide, solvent (employed in adhesives, stain removers and household cleaners), etc. [40]. Most limonene currently on the market is d-limonene (mainly technical grade, with 95 wt% purity), obtained as a by-product of citric fruit juice production through citrus peel cold-press followed by centrifugation or by steam distillation followed by condensation [24, 40, 42–47]. However, limonene availability, quality and price (3–11 $/kg [24], with an average of 7 $/kg) fluctuate substantially due to fruit bacterial disease infections and pesticides pollution [48].

**Table 1:** US market data and properties of selected terpenes

Terpene	Formula	Market price ($/kg)	Market volume (10^6^ kg/year)	Market size (10^6^ $/year)	Max. yield (g product/g glucose)	Density (g/L)	Solubility (g/L, 25 °C)	Boiling point (°C, 1 atm)
Isoprene	C_5_H_8_	1.2	173	208	0.32	681	0.3	34
d-Limonene	C_10_H_16_	7	23	160	0.32	842	Insoluble	176
β-Pinene	C_10_H_16_	5	20	102	0.32	868	Insoluble	167
α-Pinene	C_10_H_16_	2.5	22	56	0.32	860	Insoluble	155
Linalool	C_10_H_18_O	4.5	8.6	39	0.37	863	0.7	198
Squalene	C_30_H_50_	34	0.5	17	0.32	858	Insoluble	429
γ-Bisabolene	C_15_H_24_	160	< 0.5	< 73	0.32	890	Insoluble	261
Lycopene	C_40_H_56_	100	< 0.5	< 50	0.33	889	Insoluble	661
α-Humulene	C_15_H_24_	50	< 0.5	< 23	0.32	889	Insoluble	276
Valencene	C_15_H_24_	50	< 0.5	< 23	0.32	916	Insoluble	271
3-Carene	C_10_H_16_	45	< 0.5	< 23	0.32	867	Insoluble	169
γ-Terpinene	C_10_H_16_	35	< 0.5	< 16	0.32	849	Insoluble	182

The market price and volume estimates are based on the ICIS [49], CDAT [50], Alibaba.com, and IUR [51] databases. Limited volume data are available for low-volume chemicals, so only an upper bound is presented. The market size is the price multiplied by the market volume

Microbial production of limonene poses great potential in addressing such issues and thus has been the focus of many studies [16, 48, 52–61]. Several downstream separation methods have also been reported on laboratory scale, including culture extraction, solvent overlay, solid-phase micro-extraction, adsorbent polydimethylsiloxane bar, and continuous headspace removal using a cold trap [43, 60, 62–65]. However, studies on large-scale separation process synthesis and assessment of the entire production process have been limited [37]. In addition, a systematic analysis on a counterintuitive experimental phenomenon is still lacking: terpenes such as limonene are often found to be 100% present in the vapor phase after bio-conversion, despite limonene’s normal boiling point being 176 °C and the reactor operating temperature being only ~ 30 °C. Therefore, in this work, we analyze the vapor–liquid equilibrium for systems containing terpenes, synthesize alternative processes for microbial terpenes production, and perform techno-economic assessment, thereby identifying major cost drivers and key insights for all alternative process configurations.

The outline of this paper is as follows. In the “Methods” section, we discuss the bio-conversion process and analyze the vapor–liquid equilibrium and its implications on downstream separations. In the “Results and discussion” section, we present three process configurations, which are demonstrated using limonene and perform economic assessment. This is followed by an expanded study, where the costs and the corresponding process configurations in the entire feasible space, defined in terms of three key process parameters, are analyzed. Finally, we generalize our discussion to account for other terpenes, microbes, bio-conversion systems, ways to address terpene toxicity on microbes, and cellulosic biomass as a feedstock.

## Methods

The entire microbial terpene production process consists of upstream bio-conversion and downstream separations. We assume 40 T/h (“T” = metric ton) glucose supplied to the bio-conversion system (which can involve multiple fermenters in parallel), where a terpene product is produced by a microbe such as *E. coli* or *S. cerevisiae*. We choose a rate of 40 T/h because this is the amount of sugar produced by hydrolyzing 2000 T/day biomass in the NREL study [66], which we use as the basis of our bio-refinery capacity. Our goal is to obtain a technical grade terpene product (e.g., ≥ 95 wt% for limonene) after downstream separations.

### Bio-conversion process

**Fig. 1:**
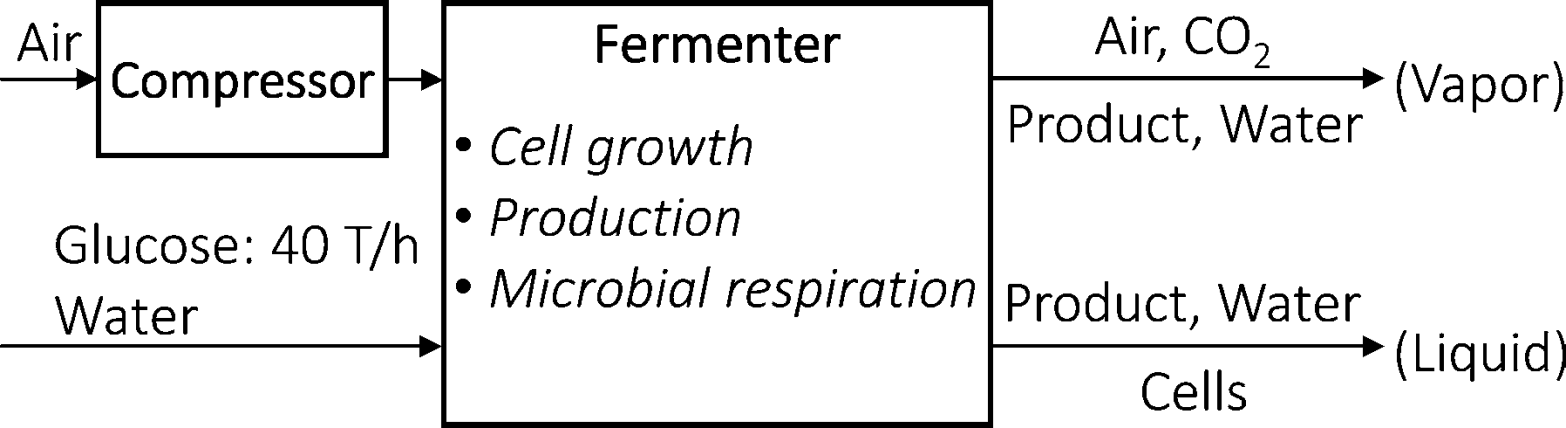
Bio-conversion process of terpene production, with 40 T/h glucose supply

Glucose, water, and air are fed into the fermenter, assumed to be operated at 30 °C and 1 atm, as shown in Fig. 1. We assume three major reactions [61, 66–68]: cell growth (Eq. 1), terpenes production (Eq. 2, with limonene as an example), and microbial respiration (Eq. 3).

As a case study, we consider limonene (C_10_H_16_) production using *E. coli* (CH_1.77_O_0.49_) [61].

1 $${\text{C}}_{6} {\text{H}}_{12} {\text{O}}_{6} \to 6{\text{CH}}_{1.77} {\text{O}}_{0.49} + 0.69{\text{H}}_{2} {\text{O}} + 1.19{\text{O}}_{2} \quad \left( {x \%\;{\text{extent}}} \right)$$

2 $$7{\text{C}}_{6} {\text{H}}_{12} {\text{O}}_{6} \to 3{\text{C}}_{10} {\text{H}}_{16} + 12{\text{CO}}_{2} + 18{\text{H}}_{2} {\text{O}}\quad \left( {y {\text{\% extent}}} \right)$$

3 $${\text{C}}_{6} {\text{H}}_{12} {\text{O}}_{6} + 6{\text{O}}_{2} \to 6{\text{H}}_{2} {\text{O}} + 6{\text{CO}}_{2} \quad \left( {z\% {\text{ extent}}} \right)$$

where Eq. 1 is adopted from the NREL study for *Z. mobilis* [66] and modified for *E. coli*, and the reaction extents are with respect to glucose consumptions. In Willrodt et al. [61], after 45 h of aerated fermentation with 14 wt% glucose concentration in the feed, glucose is depleted, while the concentrations of *E. coli* cells and limonene (extracellular) reach their maximum, and no co-products are found. The cell growth extent is ~ 46.8% (i.e., *x *= 46.8). The glucose-to-limonene yield is ~ 0.45% of the theoretical stoichiometric maximum yield of 0.32 g limonene/g glucose (i.e., limonene production extent is 0.45%; *y *= 0.45). The respiration extent is ~ 52.75% (i.e., *z *= 52.75).

For the discussions hereafter, we assume that glucose is always depleted after fermentation, i.e., *x *+ *y*+*z *= 100. Microbial respiration (Eq. 3) is the major energy source for cell growth (Eq. 1) [67, 68]. In Willrodt et al. [61], *x*:*z *= 1:1.13, which is assumed to be a fixed ratio in our current work, and thus the yield (*y* %) uniquely determines the other reaction extents as shown in Eq. 4. The ratio can be readily adjusted to account for different microbes, products, etc.

4 $$x\, = \,\frac{100\, - \,y}{2.13},\quad z\, = \,\frac{100 - y}{1.88}$$

Also note that enough air has to be supplied to meet the oxygen requirement of Eq. 3. The minimum amount of air required ($q_{\text{air}}^{\text{MIN}} , {\text{L}}$ air/g glucose) is given in Eq. 5. A common parameter describing air supply is aeration rate VVM (min^−1^)—volume of air per volume of liquid per minute, which thus has a lower bound (VVM^MIN^) to satisfy the air requirement. See the deduction and expressions of $q_{\text{air}}^{\text{MIN}}$ and VVM^MIN^ in “Background” section of Additional file 1.

5 $$q_{\text{air}}^{\text{MIN}} \, = \,0.0167\, \times \,\left( {100\, - \,y} \right)$$

The supply of nitrogen and phosphorus nutrients (e.g., using diammonium phosphate) for cell growth is neglected here because the cost is negligible (less than 1% of the total operating cost in the NREL study). Also, we assume continuous operation of the fermenter (i.e., chemostat). Experimental data obtained based on batch or fed-batch reactors (e.g., in kg) are converted into the equivalent data for chemostats (e.g., in kg/h) using the methods discussed in “Methods” section of Additional file 1.

The simulation of the entire process, including downstream separation; mass and energy balance calculations; and economic assessment, is all performed in SuperPro Designer [69] with built-in techno-economic parameters (see the specific values in Additional files 1 and 2). The split fractions calculated based on the discussed VLE calculation methods (see implementation in Additional file 2) are imported as fixed parameters into SuperPro to help specify the component flowrates after fermentation.

### Vapor–liquid equilibrium (VLE) analysis

**Fig. 2:**
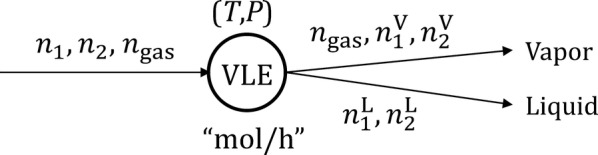
VLE analysis of immiscible liquids with inert gas. The circle marked with “VLE” represents the VLE analysis module, i.e., Eqs. 6–11

Limonene is liquid at the standard conditions, with a normal boiling point of 176 °C. However, an interesting yet counterintuitive phenomenon is reported in many studies [48, 53, 54, 57, 58, 60, 61, 64, 65]: 100% of limonene evaporates into the vapor phase after fermentation (operating at ~ 30 °C). To understand this phenomenon, we first examine the vapor–liquid equilibrium of a general immiscible liquid mixture of Components 1 (e.g., limonene) and 2 (e.g., water) with inert gas (such as air) [70]. The system is shown in Fig. 2 and described by Eqs. 6–11, where $n_{1} , n_{2}$ and $n_{\text{gas}}$ are the total molar flowrates of 1, 2 and gas; $n_{1}^{\text{V}} {\text{and}} n_{2}^{\text{V}}$ are the molar flowrates of 1 and 2 in the vapor phase; $n_{1}^{\text{L}} {\text{and }}n_{2}^{\text{L}}$ are the molar flowrates of 1 and 2 in the liquid phase. Further, we assume that the amount of gas dissolved in the liquid is negligible, which is a common assumption in such calculations; *T* and *P* are temperature and pressure; $P_{1}^{\text{T}} , P_{2}^{\text{T}}$ and $P_{\text{gas}}^{\text{T}}$ are the partial vapor pressures of 1, 2 and gas at temperature *T*; $P_{1}^{0} {\text{and }}P_{2}^{0}$ are the vapor pressures of pure 1 and 2 at temperature *T*.

Equations 6 and 7 account for material balances.

6 $$n_{1}^{\text{V}} \, + \,n_{1}^{\text{L}} \, = \,n_{1}$$

7 $$n_{2}^{\text{V}} + n_{2}^{\text{L}} = n_{2}$$

Equation 8 represents the ideal gas law.

8 $$\frac{{P_{\text{gas}}^{\text{T}} }}{{n_{\text{gas}} }} = \frac{{P_{1}^{\text{T}} }}{{n_{1}^{\text{V}} }} = \frac{{P_{2}^{\text{T}} }}{{n_{2}^{\text{V}} }}$$

Equation 9 describes the partial vapor pressures of immiscible liquids, where the pressures are independent of the mixture concentration (unlike miscible liquids described by Raoult’s law).

9 $$P_{1}^{\text{T}} \, = \,P_{1}^{0} ; \;P_{2}^{\text{T}} \, = \,P_{2}^{0}$$

Equation 10 describes the vapor pressures as functions, *f* and *g*, of temperature through, for example, Antoine equation.

10 $$P_{1}^{0} \, = \,f\left( T \right);\;P_{2}^{0} \, = \,g\left( T \right)$$

Equation 11 represents Dalton’s law.

11 $$P_{1}^{\text{T}} + P_{2}^{\text{T}} + P_{\text{gas}}^{\text{T}} = P$$

Given *n*_1_, *n*_2_, *n*_gas_, *T*, *P*, *f* and *g*, we can solve Eqs. 6–11 to obtain $n_{1}^{\text{V}}$, $n_{2}^{\text{V}}$, $n_{1}^{\text{L}}$, and $n_{2}^{\text{L}}$, describing the distribution of Components 1 and 2 in the vapor and liquid phases. For example, for $n_{1}^{\text{V}}$ we obtain

12 $$n_{1}^{\text{V}} \, = \,\frac{{f\left( T \right)\, \times \,n_{\text{gas}} }}{P\, - \,f\left( T \right)\, - \,g\left( T \right) }$$

Which monotonically increases with $n_{\text{gas}}$. However, note that based on this equation, $n_{\text{gas}}$ can be so large that $n_{1}^{\text{V}} > n_{1}$ (and thus $n_{1}^{\text{L}} < 0$ based on Eq. 6), which is physically impossible. In fact, after $n_{\text{gas}}$ exceeds the threshold value where all of Component 1 is stripped to the vapor phase, $P_{1}^{\text{T}} = P_{1}^{0}$ in Eq. 6 becomes invalid (because no liquid Component 1 exists any more). Accordingly, Eq. 12 no longer holds, and instead, $n_{1}^{\text{V}} = n_{1}$ holds. A detailed explanation can be found in Figure S2 of Additional file 1. Therefore, to account for systems where $n_{\text{gas}}$ exceeds the threshold value for Component 1, we need to (1) remove $P_{1}^{\text{T}} = P_{1}^{0}$ in Eq. 9, and (5) add $n_{1}^{\text{V}} = n_{1}$. A similar argument holds for Component 2. Thus, variables $n_{1}^{\text{V}}$, $n_{2}^{\text{V}}$, $n_{1}^{\text{L}}$ and $n_{2}^{\text{L}}$ are obtained by accounting for $n_{\text{gas}}$ in its full range, as shown in Eqs. 13–16. The detailed derivation can be found in the “Results and discussion” section of Additional file 1.

13 $$n_{1}^{\text{V}} = { \hbox{min} }\left( {\frac{{f\left( T \right) \times n_{\text{gas}} }}{P - f\left( T \right) - g\left( T \right) },\;\frac{{f\left( T \right) \times \left( {n_{\text{gas}} + n_{2} } \right)}}{P - f\left( T \right) },\;n_{1} } \right)$$

14 $$n_{2}^{\text{V}} = { \hbox{min} }\left( {\frac{{g\left( T \right) \times n_{\text{gas}} }}{P - f\left( T \right) - g\left( T \right) },\;\frac{{g\left( T \right) \times \left( {n_{\text{gas}} + n_{1} } \right)}}{P - g\left( T \right) },\;n_{2} } \right)$$

15 $$n_{1}^{\text{L}} = n_{1} - { \hbox{min} }\left( {\frac{{f\left( T \right) \times n_{\text{gas}} }}{P - f\left( T \right) - g\left( T \right) },\;\frac{{f\left( T \right) \times \left( {n_{\text{gas}} + n_{2} } \right)}}{P - f\left( T \right) },\;n_{1} } \right)$$

16 $$n_{2}^{\text{L}} = n_{2} - { \hbox{min} }\left( {\frac{{g\left( T \right) \times n_{\text{gas}} }}{P - f\left( T \right) - g\left( T \right) },\;\frac{{g\left( T \right) \times \left( {n_{\text{gas}} + n_{1} } \right)}}{P - g\left( T \right) },\;n_{2} } \right)$$

### VLE analysis in the fermenter

**Fig. 3:**
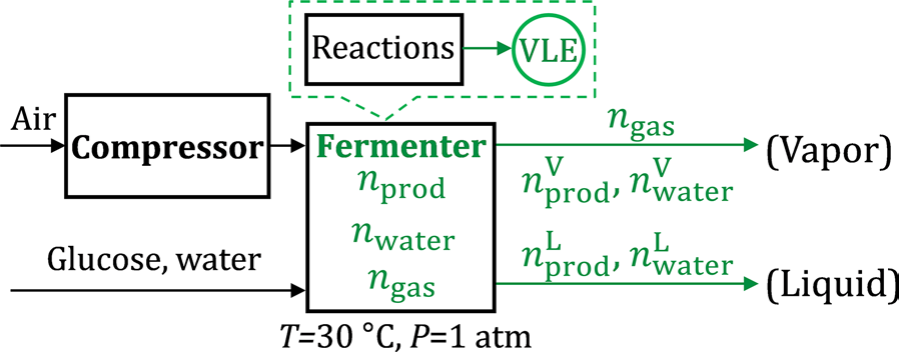
VLE analysis applied in the fermenter. Parts related to the VLE analysis are marked in green

Next, we apply the VLE analysis in the fermenter (Fig. 3). Essentially, through Eqs. 1–3, the raw materials (glucose, water and oxygen) are converted to a mixture of product, water, and gas (air and CO_2_) with flowrates $n_{\text{prod}}$, $n_{\text{water}}$, and $n_{\text{gas}}$, respectively. Based on Eqs. 13–16, we can identify the component flowrates in the vapor ($n_{\text{prod}}^{\text{V}}$ and $n_{\text{water}}^{\text{V}}$) and liquid ($n_{\text{prod}}^{\text{L}}$ and $n_{\text{water}}^{\text{L}}$). Specifically, we replace Components 1 and 2 with “product” and “water” and specify *T *= 30 °C, *P *= 1 atm, as well as *f* and *g* (for limonene and water, respectively) in Eqs. 13–16. We further relate $n_{\text{prod}}$, $n_{\text{water}}$, and $n_{\text{gas}}$ to the reactions in the fermenter and thus express them as functions of three easily comprehensible and controllable process parameters: (1) *glucose concentration* in the feed (wt%), (2) *yield* (which can be expressed either as g product/g glucose, or % of the maximum theoretical yield, i.e., *y* % in Eq. 2), and (3) *VVM* (min^−1^). The detailed deduction can be found in “Conclusions” section of Additional file 1. We find that these three parameters uniquely determine the system shown in Fig. 3.

Further, we calculate two variables that influence the downstream separation process: product split fraction to vapor (*α*) and liquid product titer (*β*, e.g., g/L), as shown in Eqs. 17 and 18, respectively.

17 $$\alpha = \frac{{n_{\text{prod}}^{\text{V}} }}{{n_{\text{prod}} }}$$

18 $$\beta = \frac{{m_{\text{prod}}^{\text{L}} }}{{Q^{\text{L}} }}$$

where $m_{\text{prod}}^{\text{L}}$ is the mass flowrate of product in the liquid phase and $Q^{\text{L}}$ is the volumetric flowrate of the liquid phase. With further deduction (“Conclusions” section of Additional file 1), we find that *α* and *β* are also functions of glucose concentration, yield and VVM.

### VLE analysis in the condenser

**Fig. 4:**
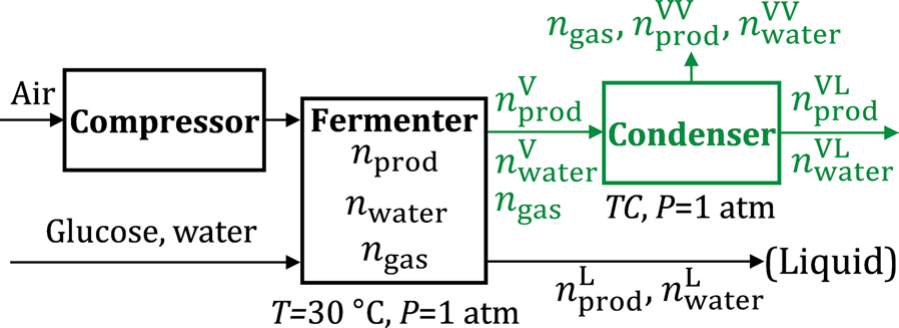
VLE analysis applied in the condenser. Parts related to the VLE analysis are marked in green. Note that $n_{\text{prod}}^{\text{V}}$ and $n_{\text{water}}^{\text{V}}$ can be calculated from the VLE analysis in the fermenter in Fig. 3

Clearly, *α* will affect the downstream separations. If *α* is large (most product goes to the vapor phase after fermentation), we should first perform a condensation (assuming at 1 atm) to convert the vapor product into liquid for further separation. To this end, as shown in Fig. 4, we perform VLE analysis in the condenser to identify $n_{\text{prod}}^{\text{VV}}$ and $n_{\text{water}}^{\text{VV}}$ in the vapor, $n_{\text{prod}}^{\text{VL}}$ and $n_{\text{water}}^{\text{VL}}$ in the condensed liquid, as well as the fraction of product condensed (*λ*) and liquid product concentration after condensation ($\omega$), as shown in Eqs. 19 and 20, respectively.

19 $$\lambda = \frac{{n_{\text{prod}}^{\text{VL}} }}{{n_{\text{prod}}^{\text{V}} }}$$

20 $$\omega = \frac{{m_{\text{prod}}^{\text{VL}} }}{{Q^{\text{VL}} }}$$

where $m_{\text{prod}}^{\text{VL}}$ is the mass flowrate of product in the condensed liquid and $Q^{\text{VL}}$ is the volumetric flowrate of the condensed liquid. With further deduction (Sect. 5 in Additional file 1), we find that *λ* and $\omega$ are also functions of glucose concentration, yield and VVM.

To maximize product recovery, we operate the condenser such that *λ *= 100% (all the product is condensed), and the condenser temperature *TC* can be calculated accordingly (see Sect. 5 of Additional file 1). For limonene, we calculate TC ≅ 0 °C.

### Case studies

We apply the VLE analysis (in both the fermenter and condenser) on three case studies (see Table 2). Specifically, Case 1 is designed based on Willrodt et al.’s data [61]. In Case 2, we assume glucose concentration and VVM values often reported in the literature, as well as a yield that may be achieved in the foreseeable future. Case 3 is designed based on the NREL study (optimistic case). Note that the O_2_ requirement is satisfied (with large enough VVM) in all three cases (see verifications in “Background” section of Additional file 1). Specifically, for the glucose concentration and yield values in Cases 1–3, VVM^MIN^ = 0.090, 0.040, and 0.0079 min^−1^, respectively.

**Table 2:** Parameters assumed for the three case studies

	Glucose concentration (wt%)	Yield (g product/g glucose)	Yield (% of max.)	VVM (min^−1^)	Residence time (h)
Case 1	14	0.00144	0.45	0.1	45
Case 2	10	0.122	38	0.1	45
Case 3	24	0.304	95	0.01	45

**Fig. 5:**
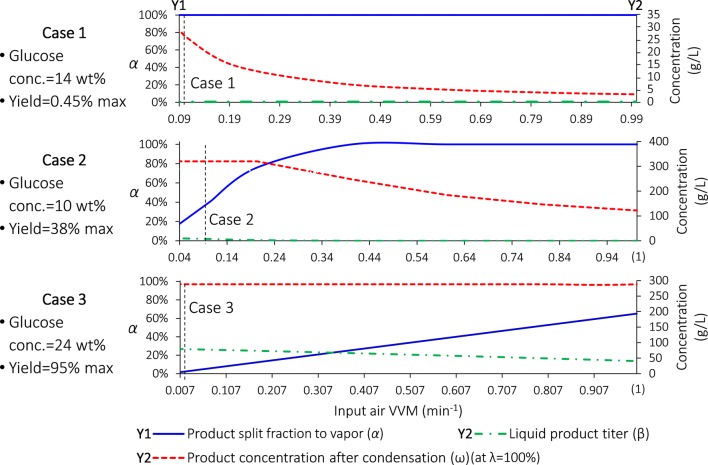
*α*, *β,* and *ω* as functions of VVM. Note that VVM^MIN^ = 0.090, 0.040, and 0.0079 min^−1^ for Cases 1–3, respectively. C*onc*. concentration

Variables *α*, *β,* and ω (at *λ *= 100%) with varying VVM for the three cases are shown in Fig. 5. The specific parameters and calculations can be found in Additional files 1 and 2. Clearly, by increasing VVM, *α* increases while *β* and ω decrease. Note that, based on the “Conclusions” section of Additional file 1, larger *α* will be achieved with lower glucose concentration, lower yield and higher VVM. For this reason, we note the following. First, *α *= 100% (all the product goes to the vapor phase after fermentation) in Case 1 mainly due to very low yield, which explains the common experimental phenomenon that all the limonene ends up in the vapor phase. Second, *α *= 35.1% in Case 2. Third, *α *= 1.7% in Case 3 due to high glucose concentration, high yield and low VVM.

### VLE implications on downstream separation

**Fig. 6:**
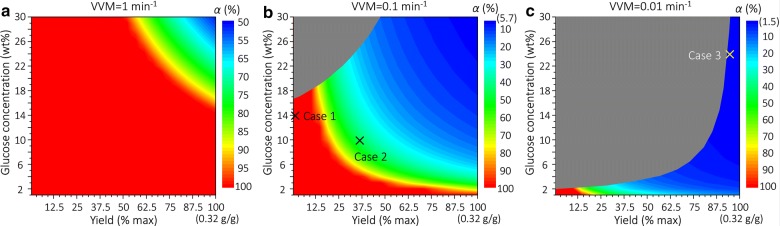
Heat maps showing the influence of glucose concentration and yield on *α* at different VVM values. **a** VVM = 1 min^−1^; **b** VVM = 0.1 min^−1^; **c** VVM = 0.01 min^−1^. The numbers in parentheses on the color scales in **b** and **c** denote the corresponding minimum *α* values. Gray-shaded areas represent infeasible regions where VVM < VVM^MIN^ (calculated using the method discussed in “Bio-conversion process” section)

While all the four process variables (*α*, *β*, *λ* and ω) influence separation cost, it is *α* that determines the suitable separation process configurations. The heat diagrams depicting the influence of glucose concentration and yield on *α* at different VVM values (1, 0.1 and 0.01 min^−1^) are shown in Fig. 6. The downstream separation process should be synthesized accordingly. Specifically, when *α* is very large (e.g., 99.9%), only the product in the vapor phase after fermentation needs recovery, and thus a condenser is first used to condense the vapor stream (as discussed in “VLE analysis in condenser” section), followed by centrifugal decantation that separates the product (obtained as a top oil phase) from water. When *α* is very small (e.g., 1%), all the product is practically in the liquid stream, so a direct centrifugal decantation suffices. Analyses based on several general bio-separation process synthesis methods [71–77], where different separation technologies are compared, also support the use of centrifugal decantation for this class of products. When *α* is neither very large nor very small, the product in both the vapor and liquid phases needs to be recovered.

## Results and discussion

### Alternative process configurations

**Fig. 7:**
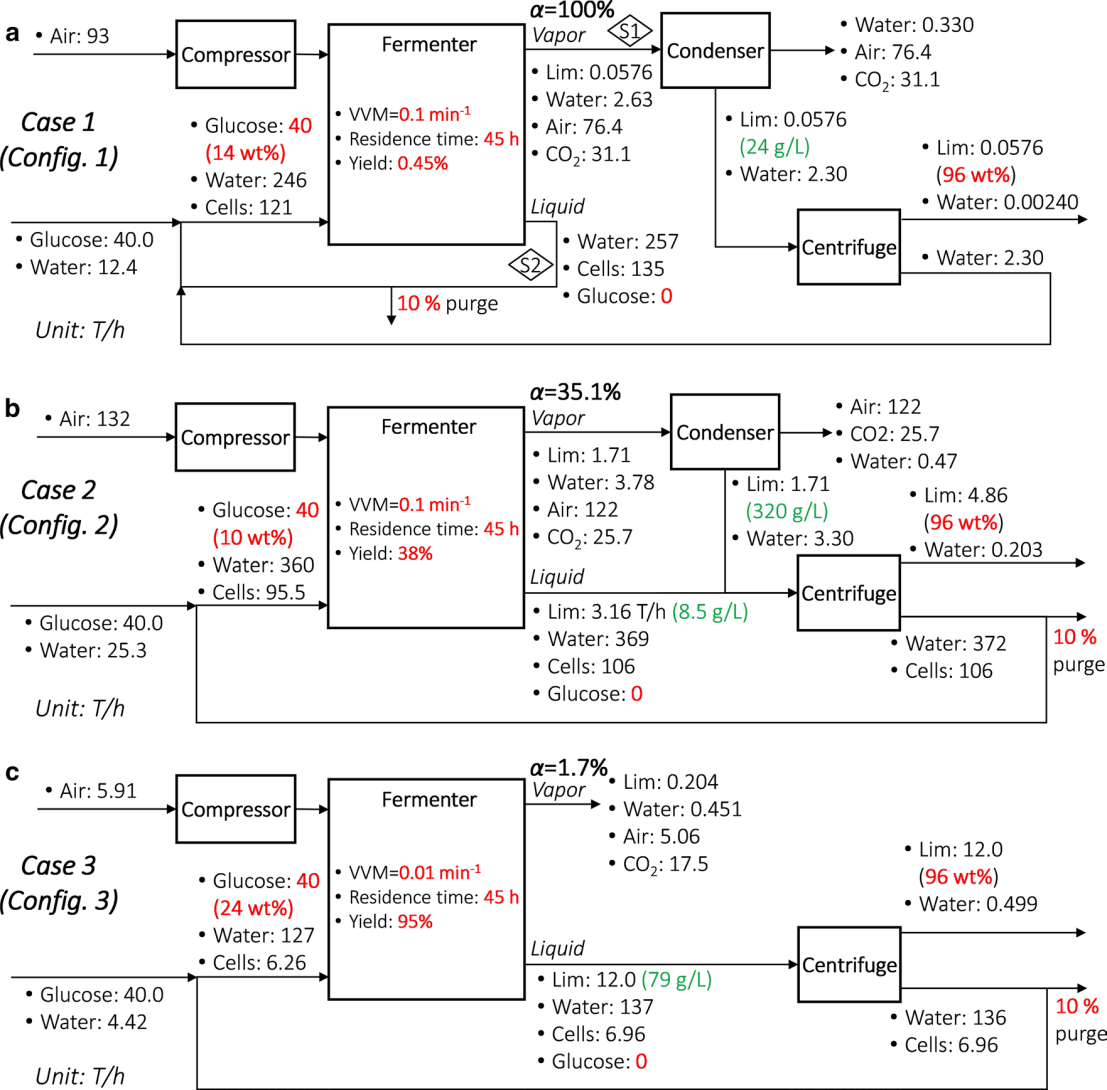
Three process configurations. **a** Configuration 1 (e.g., Case 1), where the product is mainly in the vapor; **b** Configuration 2 (e.g., Case 2), where the product is distributed in the vapor and liquid; **c** Configuration 3 (e.g., Case 3), where the product is mainly in the liquid. Given parameters for each case are marked bold in red. *Lim* limonene. For the component flowrates in each stream, the units are T/h, and three significant figures are kept. Product concentrations in specific streams are marked in green

The process configurations suitable for different *α* values are shown in Fig. 7. They also correspond to Cases 1–3 as examples. The liquid stream (S2), either with or without centrifugation, is recycled to save on water cost, and a 10% purge ratio is adopted to avoid accumulation of microbial cells. For the centrifugal decantation, we assume the limonene oil globule diameter to be 20 μm [78]. It is used to calculate the rising velocity of limonene oil globules, which impacts the equipment size and electricity consumption. Finally, a 96 wt% limonene product stream is obtained after centrifugation. Key parameters and component flowrates are shown in Fig. 7. Other parameters for each unit can be found in Additional file 1. Also note the subtle trade-off between Configurations 1 and 2 when *α* is very large: sending the product in the liquid stream (S2) also to the centrifuge (as in Configuration 2) reduces product loss (compared with Configuration 1) but at the same time increases the centrifuge input flowrate (thus larger equipment and cost). Therefore, both configurations can be applicable in specific cases depending on the trade-off.

### Economic assessment of the case studies

**Fig. 8:**
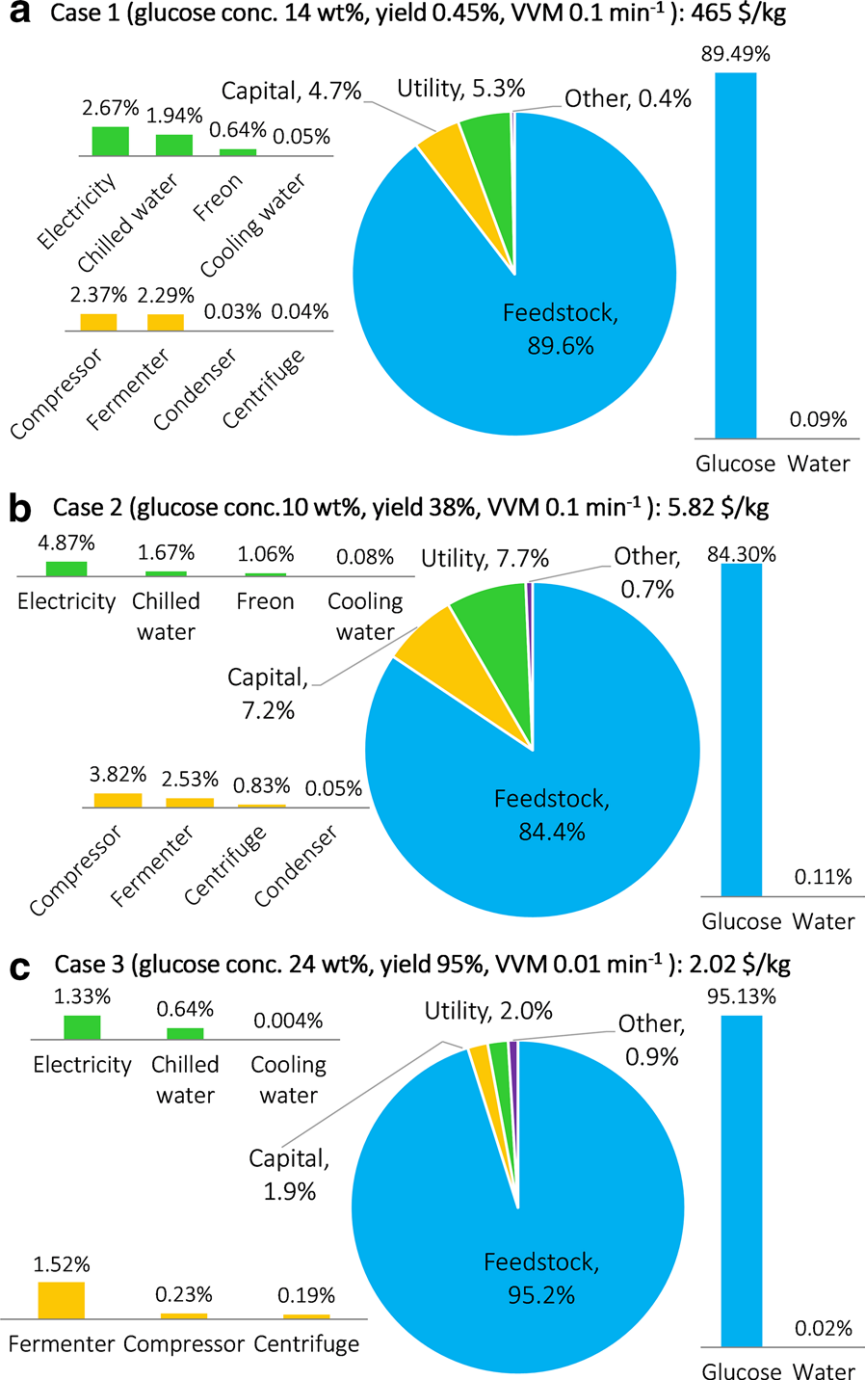
Cost distribution by cost types. **a** Case 1; **b** Case 2; **c** Case 3. Capital costs are annualized

The costs for the three cases, by cost types, are summarized in Fig. 8. Both capital cost and operating costs, including costs of feedstock, utility, and other (labor and miscellaneous), are included. Costs by units are summarized in Sect. 6 of Additional file 1. The total unit production costs for the three cases are 465, 5.82, and 2.02 $/kg limonene, respectively. Feedstock is the major cost component in all cases. In comparison, lower cost is achieved with a higher glucose concentration (thus smaller equipment size and therefore lower capital and utility costs), a higher yield (thus high product flowrate and therefore lower unit production cost), and a lower VVM (thus cheaper air compression).

Unlike many other bio-based chemical production processes, where separation accounts for 60–80% of the total cost [13, 79], separation is not the major cost driver here mainly because limonene (and most other terpenes) is extracellular, insoluble, and lighter than water (in terms of density), which requires a simple centrifugal decantation. Note that the separation cost will increase when a product’s oil globule size is smaller and the density is closer to that of water (based on Stokes’s law), which can lead to a smaller globule rising velocity in the decanter and thus larger equipment. For example, if we consider a terpene with globule diameter 1 μm and density 950 g/L, instead of limonene (20 μm and 841 g/L), then the centrifugation cost in Case 3 will increase by 40% (although the total cost will increase by just 0.1% due to high feed glucose cost).

**Fig. 9:**
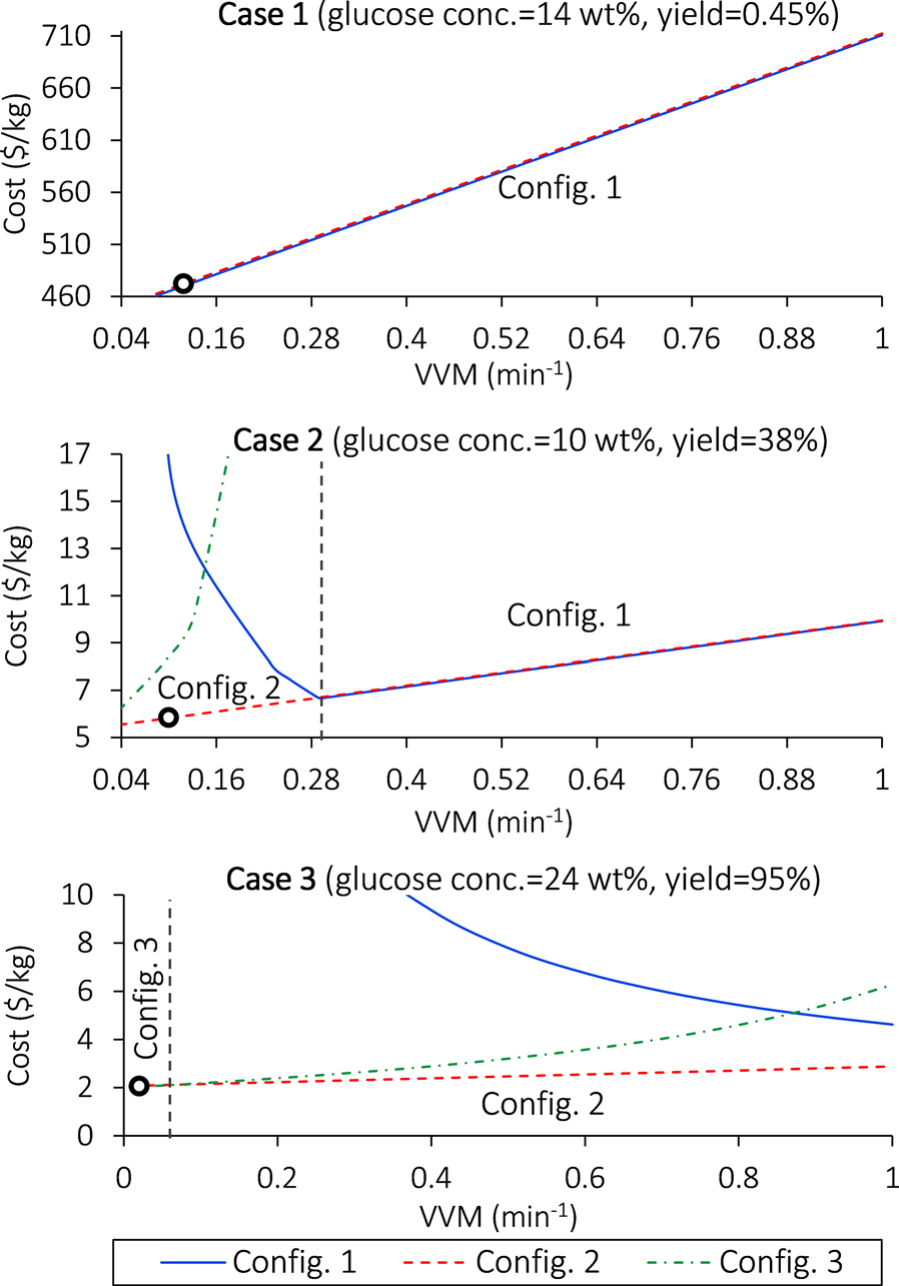
Cost comparison between configurations with varying VVM values in different cases. The vertical dashed lines denote threshold VVM values where the cost-minimal configurations change; the optimal configurations are labeled in the corresponding regions. The three cases are represented by the small circles. Note that VVM^MIN^ = 0.090, 0.040, and 0.0079 min^−1^ for Cases 1–3, respectively

To compare configurations under different glucose concentration, yield, and VVM, we generate Fig. 9, where the optimal configurations are labeled in the corresponding regions. In general, Configuration 2 is a low-cost option, but under specific conditions, the other configurations can have lower costs.

### General economic assessment

**Fig. 10:**
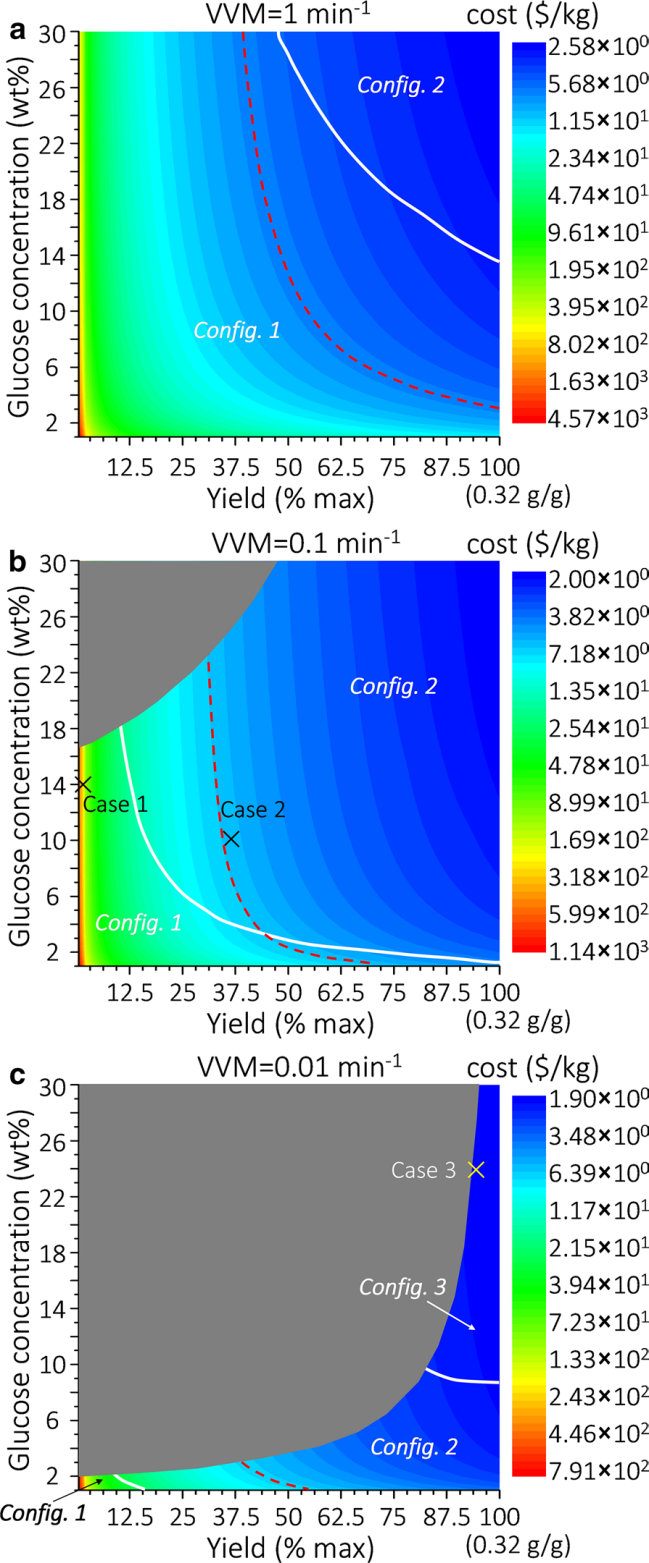
Heat maps depicting the influence of glucose concentration and yield on configurations and costs. **a** VVM = 1 min^−1^; **b** VVM = 0.1 min^−1^; **c** VVM = 0.01 min^−1^. The color scales are plotted logarithmically. The white solid contour curves denote the boundaries where the optimal process configurations change. The red dashed curves denote break-even combinations of glucose concentration and yield. Gray-shaded areas represent infeasible regions where VVM < VVM^MIN^. Note that 50% yield is likely a reasonable target in the foreseeable future

To identify the cost-minimal process configuration and the corresponding cost, for any case, we can perform economic assessment assuming that all the three configurations can be used and then choose the one with the minimum cost. We perform such analysis for various glucose concentration and yield combinations under three VVM values (1, 0.1, and 0.01 min^−1^), as represented in the “heat maps” in Fig. 10a–c, respectively. The color scales represent the minimum unit production costs, and the optimal process configurations are marked accordingly in the different regions separated by the white contour curves. Gray-shaded areas represent infeasible regions where VVM < VVM^MIN^. Clearly, Configuration 1 is optimal at relatively high VVM, low glucose concentration, and low yield (rendering high cost); Configuration 3 is optimal at relatively low VVM, high glucose concentration, and high yield (rendering low cost); Configuration 2 is optimal in between. The cost-price break-even cases (cost = 7 $/kg) are also shown, marked with red dashed curves. Also note that we present yield up to 100%, but 50% is likely a reasonable target in the foreseeable future.

It is also worth noting that evaporation of limonene actually generates a more concentrated product stream after condensation (e.g., 26 g/L after condensation vs. 0.22 g/L assuming no evaporation in the fermenter in Case 1; see Fig. 7a), which facilitates downstream separation. However, we should not increase VVM to “push” more product to the vapor phase, because a higher VVM for each glucose concentration and yield combination in Fig. 10 leads to a higher cost. In other words, the additional costs of compression and condensation due to increased VVM outweigh the savings from separation (centrifugal decantation).

### Addressing toxicity: use of solvent vs. microbial engineering

**Fig. 11:**
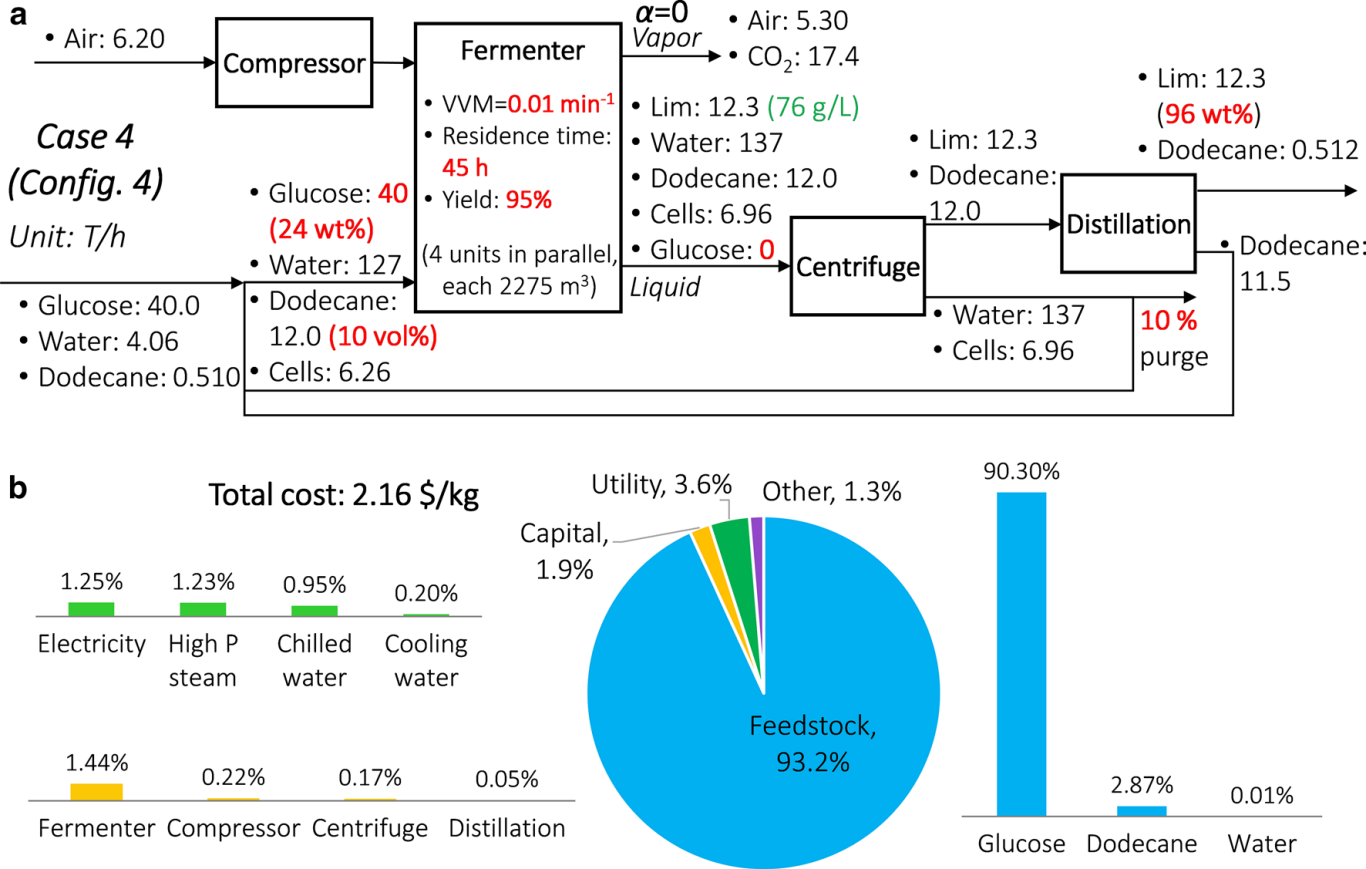
Process configuration and economic assessment of Case 4. **a** Process Configuration 4; **b** cost distribution

Some terpenes like limonene are, in fact, toxic to microbes, which prevents product titer from reaching a high level. There are currently two major methods to address this challenge: (1) engineering of the microbes to increase resilience, and (2) biphasic fermentation, including air stripping of the product and the use of a solvent miscible with the product (and immiscible with water) to reduce terpene concentration in the aqueous phase, where the microbes exist [40]. The first method has been assumed in our discussions so far when Configurations 2 and 3 are used, thus allowing the product yield to reach a high level. Also, using a chemostat helps further alleviate the problem because products and microbes are constantly withdrawn from the fermenter. In the second method, air stripping is used in Configuration 1, where the product flows to the vapor phase while the microbes stay in the liquid phase. The use of solvent (such as dodecane, commonly used for limonene) can reduce terpene concentration in the aqueous phase, while simultaneously preventing evaporation [40, 48, 61]. Also, the use of solvent can potentially help remove any additional impurities due to the lysis of cells. We develop a process based on such solvent-based separation (Configuration 4). It is demonstrated using Case 4 as shown in Fig. 11a.

Specifically, we adopt the parameters from Case 3 (glucose concentration = 24 wt%, yield = 95%, and VVM = 0.01 min^−1^) and add dodecane as a solvent (miscible with limonene and immiscible with water) into the fermenter. Since dodecane prevents evaporation, only the liquid stream is treated with centrifugal decantation. The limonene–dodecane mixture is then distilled to obtain the final product, while dodecane is recycled. In terms of the amount of dodecane used, values between 5 and 20 vol% are reported in the literature [54, 60, 80, 81]. We assume 10 vol% here.

**Fig. 12:**
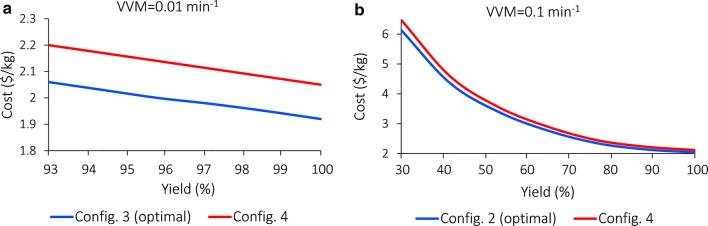
Cost comparison between Configuration 4 and the non-solvent configuration. **a** Glucose concentration = 24 wt% and VVM = 0.01 min^−1^; **b** glucose concentration = 24 wt% and VVM = 0.1 min^−1^. Only the yield ranges that satisfy VVM > VVM^MIN^ are shown

The total cost is 2.16 $/kg, as shown in Fig. 11b. Costs by units are summarized in Sect. 7 of Additional file 1. Cost comparisons with the non-solvent scenario (microbial engineering) under glucose concentration = 24 wt% and VVM = 0.01 and 0.1 min^−1^, respectively, are shown in Fig. 12a, b. When VVM = 0.01 min^−1^, Configuration 4 is ~ 0.14 $/kg (7%) more expensive than Configuration 3 (the optimal configuration assuming no use of solvent); when VVM = 0.1 min^−1^, Configuration 4 is 0.08–6.48 $/kg (4–6%) more expensive than Configuration 2 (optimal).

### Extensions

The method presented in this work can be used to study systems producing different terpenes (by modifying Eq. 2 and product physical properties accordingly), as well as different microbes (by modifying Eq. 1) and bio-conversion systems (by replacing the fermenter with, for example, an open pond, and glucose with CO_2_ to account for photosynthesis instead of fermentation).

We study production systems for different terpenes using the assumptions for Case 3 (glucose concentration = 24 wt%, yield = 95%, VVM = 0.01 min^−1^). Cost estimates along with the expected annual profit assuming 100% market share for all the terpenes in Table 1 using the methods discussed in this work are presented in Table 3. Also, the break-even yield for Configurations 1–3 is calculated for each product, assuming Case 1 glucose concentration (14 wt%) and VVM (0.1 min^−1^). The minimum break-even yield, across configurations, and the corresponding optimal configuration are shown in Table 4.

**Table 3:** Minimum break-even yields and the corresponding optimal process configurations for 12 terpenes

Terpene	Minimum break-even yield (% max.)	Optimal configuration
Isoprene	NA	NA
d-Limonene	30	2
β-Pinene	42	2
α-Pinene	85	2
Linalool	41	2
Squalene	5.9	3
γ-Bisabolene	1.2	3
Lycopene	1.9	3
α-Humulene	4.0	3
Valencene	4.0	3
3-Carene	4.7	1
γ-Terpinene	5.9	2

Glucose concentration = 24 wt%, yield = 95%, VVM = 0.01 min^−1^

**Table 4:** Minimum break-even yields and the corresponding optimal process configurations for 12 terpenes

Terpene	Minimum break-even yield (% max.)	Optimal configuration
Isoprene	NA	NA
d-Limonene	30	2
β-Pinene	42	2
α-Pinene	85	2
Linalool	41	2
Squalene	5.9	3
γ-Bisabolene	1.2	3
Lycopene	1.9	3
α-Humulene	4.0	3
Valencene	4.0	3
3-Carene	4.7	1
γ-Terpinene	5.9	2

Glucose concentration = 14 wt% and VVM = 0.1 min^−1^ are assumed

Limonene appears to be the most promising short-term target because it has a comparatively high expected profit based on our current assumptions (Table 3), as well as a relatively low break-even yield (Table 4). For the terpenes with high prices yet low market volumes, the break-even yields are even easier to achieve, but the total market size is small. More accurate market volume data are needed to better quantify their economic prospect. Also, low-volume products are often used for niche markets (e.g., cosmetics and pharmaceuticals), which have much stricter final product requirements, and thus their actual costs may be underestimated here.

In addition, if cellulosic biomass, instead of pure glucose, can be used as the feedstock (as in the NREL process), then the process before fermentation includes biomass pre-treatment and enzymatic hydrolysis [66]. The stream after hydrolysis contains 240 g/L sugar, and the cost of pre-treatment and hydrolysis is ~ 0.28 $/kg (pure sugar basis), i.e., a 53% feedstock cost saving compared to the 0.6 $/kg pure glucose price. We do not consider the use of cellulosic biomass in this study due to its limited applications in industry. Nonetheless, the readers can account for this technology by reducing the current glucose cost by 53%.

Note that this work is intended to be a high-level synthesis and analysis of bio-based terpene processes. Our goal is to identify the key cost drivers and provide target values for the researchers working in the area. If the key parameters discussed herein are substantially improved in the future, so that a positive profit margin can potentially be achieved, then more detailed studies accounting for aspects such as contamination prevention and microbial cell growth control would be required.

Finally, we note that the approaches discussed in this work can be used to study processes for the production of other extracellular, insoluble, and light (in terms of density compared to water) products, such as fatty acids.

## Conclusions

This work focuses on the process synthesis, simulation, and techno-economic evaluation of microbial terpenes production. We first analyzed the vapor–liquid equilibrium, which explained the counterintuitive experimental phenomenon where terpenes such as limonene (normal boiling point 176 °C) are often found to be 100% present in the vapor phase after fermentation (at ~ 30 °C). Based on this analysis, we further proposed three alternative process configurations, demonstrated with three different case studies.

We estimated that the total unit production costs of the three cases are 465, 5.82, and 2.02 $/kg, respectively. We also identified the key cost drivers to be (1) feed glucose concentration, (2) yield, and (3) VVM. We further showed how these drivers impact costs and the selectin of the corresponding configurations. We found that the production of limonene, based on current literature experimental data, is economically infeasible and that higher glucose concentration and yield are key to lowering the cost.

Finally, we extended the assessment to account for different process parameters, terpene products, strategies to address terpene toxicity (microbial engineering vs. use of solvent), and cellulosic biomass as a feedstock. After studying the economics of 12 terpenes, limonene appears to be the most reasonable short-term target.

The framework and suite of methods presented herein are applicable to a wide range of extracellular insoluble chemicals with density lower than that of water, such as fatty acids. Therefore, the proposed methods can provide guidance and useful insights into the development of bio-based production systems for terpenes and other bioproducts.

## Additional files

**Additional file 1.** Additional calculations and details of the analyses.

**Additional file 2.** Editable Excel file containing (1) assumptions for TEA, (2) VLE calculations, and (3) Figures.
