# Supplementary material for: Synthesis and techno-economic assessment of microbial-based processes for terpenes production
Source: Biotechnol Biofuels. 2018 Oct 27;11:294. doi: 10.1186/s13068-018-1285-7 (PMC6203976; doi:10.1186/s13068-018-1285-7)
Supplement: Supplementary file 1 — Additional file 1. Additional calculations and details of the analyses. [file 13068_2018_1285_MOESM1_ESM.docx]

**Synthesis and Techno-Economic Assessment of Microbial-Based Processes for Terpenes Production**

**Wenzhao Wu**, **Christos T. Maravelias^*^**

Dept. of Chemical and Biological Engineering and DOE Great Lakes Bioenergy Research Center, University of Wisconsin-Madison, Madison, WI 53706

**Additional File 1**

**1. VVM^MIN^ calculation**

The minimum amount of air required ($q_{\mathrm{air}}^{\mathrm{MIN}}, L$ air/g glucose) can be calculated based on the stoichiometry of Equations 1-4 in the main text. The result is shown in Equation A1.

$q_{\mathrm{air}}^{\mathrm{MIN}}=\frac{\left( \frac{\eta\times z\%}{\zeta}-\frac{\theta\times x\%}{\zeta} \right)\times\sigma}{\mathrm{MW}_{O2}\times\nu}=\frac{\left( \frac{192\times z\%}{180}-\frac{38.08\times x\%}{180} \right)\times24 L/mol}{32g/mol\times21\% oxygen in air}=0.0167\times(100-y)$ (A1)

where *ζ*, *η* and *θ* represent the stoichiometrically weighted molecular weights of glucose and O_2_ (consumed and produced), respectively; MW_O2_ is the molecular weight of O_2_; $\sigma$ is the molar volume of gas at normal conditions; ν is the volumetric percentage of oxygen in air.

VVM^MIN^ is derived as shown in Equation A2 using the definition of VVM, where $q^{\mathrm{IN}}$ is the total volumetric flow rate of the feed liquid per unit of glucose flow rate (L/g glucose); $\tau$ is fermenter residence time (min); $\rho_{\mathrm{liq}}$ is density (g/L) of the feed (glucose-water mixture), which is a fitted function based on known experimental data points (see Additional File 2), i.e., $\rho_{\mathrm{liq}}=294.75\times glucose concentration (wt\%)+1000$.

$\mathrm{VVM}^{\mathrm{MIN}}=\frac{q_{\mathrm{air}}^{\mathrm{MIN}}}{q^{\mathrm{IN}}\times\tau}=\frac{0.0167\times(100-y)}{\frac{1}{glucose concentration\times\rho_{\mathrm{liq}}}\times45h\times60min/h}=\frac{(100-y)\times glucose concentration\times(294.75\times glucose concentration+1000)}{161677}$ (A2)

For example, in Case 1 (glucose concentration = 14 wt%, yield = 0.45 %, i.e., *y*=0.45), $\mathrm{VVM}^{\mathrm{MIN}}=0.090$ min^-1^, which is satisfied by the 0.1 min^-1^ VVM in Case 1. The same verification can be performed for Cases 2 and 3, where VVM^MIN^=0.040 and 0.0079 min^-1^, respectively.

We can also identify infeasible yield and glucose concentration combinations for a given VVM, as represented by the grey shaded areas in **Figures 6** and **10** in the main text, by fixing VVM^MIN^ to the given VVM value in Equation A2. The specific calculations can be found in Additional File 2.

**2. Batch-to-chemostat data conversion**

**Figure S1**. Conversion of data in a batch reactor (kg) to equivalent data in a chemostat (kg/h).

**Figure S1** shows how we convert data in a batch reactor to equivalent data in a chemostat.

**3. Explanation to “min” functions**

The $n_{\mathrm{gas}}$ threshold values for Components 1 and 2, where the corresponding component is completely stripped to the vapor phase, are $\frac{n_{1}\times(P-f\left( T \right)-g(T)\text{ })}{f\left( T \right)}$ and $\frac{n_{2}\times(P-f\left( T \right)-g(T)\text{ })}{g\left( T \right)}$, respectively, which are calculated with $\frac{f\left( T \right)\times n_{\mathrm{gas}}}{P-f\left( T \right)-g(T)\text{ }}=n_{1}$ and $\frac{g\left( T \right)\times n_{\mathrm{gas}}}{P-f\left( T \right)-g(T)\text{ }}=n_{2}$ by definition.

**Figure S2**. Changes to VLE with increasing n_gas_ in different ranges. (A) n_gas_ is below the threshold values for both Components 1 and 2; (B) n_gas_ is above the threshold value for Component 1 but not for Component 2; (C) n_gas_ is above the threshold values for both Components 1 and 2. It is assumed that the $n_{gas}$ threshold value for Component 1 is lower than that for Component 2, as an example here. Constants are marked bold in red. The change (increase/decrease) for each variable is marked with a green directional arrow. V represents the total volumetric flow rate of the vapor phase. The components are separated into different regions for demonstration purpose only; in reality, they are mixed together.

The qualitative VLE analysis in different *n*_gas_ ranges is shown in **Figure S2**. For demonstration purpose, we first assume that the $n_{\mathrm{gas}}$ threshold value for Component 1 is lower than that for Component 2; in other words, when Component 1 is completely stripped to the vapor, a portion of Component 2 still exists in the liquid, i.e., $\frac{n_{1}}{f\left( T \right)}<\frac{n_{2}}{g\left( T \right)}$. There are three possible scenarios as follows.

**Scenario 1**. When *n*_gas_ is below the threshold values for both Components 1 and 2 (**Figure S2-A**), the increase of *n*_gas_ leads to more Components 1 and 2 being pushed to the vapor phase.

**Scenario 2**. When *n*_gas_ is above the threshold value for Component 1 but not for Component 2 (**Figure S2-B**), $n_{1}^{V}$=$n_{1}$, i.e., no more Component 1 is left to be pushed to the vapor phase further, leading to the drop of $P_{1}^{T}$ and increase of $P_{\mathrm{gas}}^{T}$.

**Scenario 3**. When *n*_gas_ is above the threshold values for both Components 1 and 2 (**Figure S2-C**), $n_{1}^{V}$=$n_{1}$ and $n_{2}^{V}$=$n_{2}$, leading to drop of $P_{1}^{T}$and $P_{2}^{T}$ as well as increase of $P_{\mathrm{gas}}^{T}$

A quantitative analysis for the three scenarios is as follows.

In Scenario 1, $\frac{f\left( T \right)\times n_{\mathrm{gas}}}{P-f\left( T \right)-g(T)\text{ }}<n_{1}$, while $\frac{g\left( T \right)\times n_{\mathrm{gas}}}{P-f\left( T \right)-g(T)\text{ }}<n_{2}$ is implicit because $\frac{n_{1}}{f\left( T \right)}<\frac{n_{2}}{g\left( T \right)}$. Thus, $n_{\mathrm{gas}}<\frac{n_{1}\times(P-f\left( T \right)-g(T)\text{ })}{f\left( T \right)}$ uniquely describes Scenario 1. We solve Equations 3-8 in the main text to obtain the following.

$n_{1}^{V}=\frac{f\left( T \right)\times n_{\mathrm{gas}}}{P-f\left( T \right)-g(T)\text{ }}$ (A3)

$n_{2}^{V}=\frac{g\left( T \right)\times n_{\mathrm{gas}}}{P-f\left( T \right)-g(T)\text{ }}$ (A4)

In Scenario 2, $\frac{f\left( T \right)\times n_{\mathrm{gas}}}{P-f\left( T \right)-g(T)\text{ }}\geq n_{1}$ and $\frac{g\left( T \right)\times n_{\mathrm{gas}}}{P-f\left( T \right)-g(T)\text{ }}<n_{2}$, and thus $\frac{n_{1}\times(P-f\left( T \right)-g(T)\text{ })}{f\left( T \right)}\leq n_{\mathrm{gas}}<\frac{n_{2}\times(P-f\left( T \right)-g(T)\text{ })}{g\left( T \right)}$, the following equations hold (as discussed in the last paragraph of Section “VLE analysis” in the main text).

$n_{1}^{V}+n_{1}^{L}=n_{1}$ (A5)

$n_{2}^{V}+n_{2}^{L}=n_{2}$ (A6)

$n_{1}^{V}=n_{1}$ (A7)

$\frac{P_{\mathrm{gas}}^{T}}{n_{\mathrm{gas}}}=\frac{P_{1}^{T}}{n_{1}^{V}}=\frac{P_{2}^{T}}{n_{2}^{V}}$ (A8)

$P_{2}^{T}=P_{2}^{0}$ (A9)

$P_{1}^{0}=f\left( T \right);P_{2}^{0}=g\left( T \right)$ (A10)

$P_{1}^{T}+P_{2}^{T}+P_{\mathrm{gas}}^{T}=P$ (A11)

Solving Equations A5-A11, we obtain the following.

$n_{1}^{V}=n_{1}$ (A12)

$n_{2}^{V}=\frac{g\left( T \right)\times{(n}_{\mathrm{gas}}+n_{1})}{P-g(T)\text{ }}$ (A13)

In Scenario 3, $\frac{g\left( T \right)\times n_{\mathrm{gas}}}{P-f\left( T \right)-g(T)\text{ }}\geq n_{2}$, while $\frac{f\left( T \right)\times n_{\mathrm{gas}}}{P-f\left( T \right)-g(T)\text{ }}\geq n_{1}$ is implicit because $\frac{n_{1}}{f\left( T \right)}<\frac{n_{2}}{g\left( T \right)}$. Thus, $n_{\mathrm{gas}}\geq\frac{n_{2}\times(P-f\left( T \right)-g(T)\text{ })}{g\left( T \right)}$ uniquely describes Scenario 3, and we obtain the following.

$n_{1}^{V}=n_{1}$ (A14)

$n_{2}^{V}=n_{2}$ (A15)

Results for the above three scenarios (Equations A3, A4, and A12-A15) can be summarized as the following.

$n_{1}^{V}=\min(\frac{f\left( T \right)\times n_{\mathrm{gas}}}{P-f\left( T \right)-g(T)\text{ }}$, $n_{1}$) (A16)

$n_{2}^{V}=\min(\frac{g\left( T \right)\times n_{\mathrm{gas}}}{P-f\left( T \right)-g(T)\text{ }}$, $\frac{g\left( T \right)\times(n_{\mathrm{gas}}+n_{1})}{P-g(T)\text{ }}$, $n_{2}$) (A17)

To verify the validity of Equations A16 and 17, note that in Equation A17, $\frac{g\left( T \right)\times n_{\mathrm{gas}}}{P-f\left( T \right)-g(T)\text{ }}-\frac{g\left( T \right)\times\left( n_{\mathrm{gas}}+n_{1} \right)}{P-g\left( T \right)\text{ }}=\frac{g\left( T \right)\times\{f\left( T \right)\times n_{\mathrm{gas}}-n_{1}\times\left[ P-f\left( T \right)-g\left( T \right) \right]\}}{\left[ P-f\left( T \right)-g\left( T \right)\text{ } \right]\times\left[ P-g\left( T \right) \right]}$. Therefore,

(1) When $\frac{g\left( T \right)\times n_{\mathrm{gas}}}{P-f\left( T \right)-g(T)\text{ }}<n_{2}$ and $f\left( T \right)\times n_{\mathrm{gas}}-n_{1}\times\left[ P-f\left( T \right)-g\left( T \right) \right]<0$, i.e., $\frac{f\left( T \right)\times n_{\mathrm{gas}}}{P-f\left( T \right)-g(T)\text{ }}<n_{1}$, we have $\frac{g\left( T \right)\times n_{\mathrm{gas}}}{P-f\left( T \right)-g(T)\text{ }}<\frac{g\left( T \right)\times(n_{\mathrm{gas}}+n_{1})}{P-g(T)\text{ }}<n_{2}$, and thus $n_{2}^{V}=\frac{g\left( T \right)\times n_{\mathrm{gas}}}{P-f\left( T \right)-g(T)\text{ }}$ and $n_{1}^{V}=\frac{f\left( T \right)\times n_{\mathrm{gas}}}{P-f\left( T \right)-g(T)\text{ }}$, which corresponds to Scenario 1.

(2) When $\frac{g\left( T \right)\times n_{\mathrm{gas}}}{P-f\left( T \right)-g(T)\text{ }}<n_{2}$ and $f\left( T \right)\times n_{\mathrm{gas}}-n_{1}\times\left[ P-f\left( T \right)-g\left( T \right) \right]\geq0$, i.e., $\frac{f\left( T \right)\times n_{\mathrm{gas}}}{P-f\left( T \right)-g(T)\text{ }}\geq n_{1}$, we have $\frac{g\left( T \right)\times(n_{\mathrm{gas}}+n_{1})}{P-g(T)\text{ }}\leq\frac{g\left( T \right)\times n_{\mathrm{gas}}}{P-f\left( T \right)-g(T)\text{ }}<n_{2}$ $n_{2}^{V}=\frac{g\left( T \right)\times(n_{\mathrm{gas}}+n_{1})}{P-g(T)\text{ }}$ and $n_{1}^{V}=n_{1}$, which corresponds to Scenario 2.

(3) When $\frac{g\left( T \right)\times n_{\mathrm{gas}}}{P-f\left( T \right)-g(T)\text{ }}\geq n_{2}$, $\frac{f\left( T \right)\times n_{\mathrm{gas}}}{P-f\left( T \right)-g(T)\text{ }}\geq n_{1}$ always holds because $\frac{n_{1}}{f\left( T \right)}<\frac{n_{2}}{g\left( T \right)}$. Thus, $n_{2}\leq\frac{g\left( T \right)\times(n_{\mathrm{gas}}+n_{1})}{P-g(T)\text{ }}\leq\frac{g\left( T \right)\times n_{\mathrm{gas}}}{P-f\left( T \right)-g(T)\text{ }}$. Therefore, we have $n_{2}^{V}=n_{2}$ and $n_{1}^{V}=n_{1}$, which corresponds to Scenario 3.

With a similar deduction procedure, if we further account for scenarios where the $n_{\mathrm{gas}}$ threshold value for Component 2 is lower than that for Component 1, i.e., $\frac{n_{2}}{g\left( T \right)}<\frac{n_{1}}{f\left( T \right)}$, Equations 10-13. in the main text can be obtained, which accounts for all possible scenarios.

**4. VLE analysis in the fermenter**

**Figure S3**. VLE analysis applied in the fermenter. Parts related to the VLE analysis are marked in green.

To apply the VLE analysis in the fermenter, we replace Components 1 and 2 with “product” and “water”, and specify *T*=30 °C, *P*=1 atm, as well as *f* and *g* (for limonene and water, respectively). Thus, we find that the system (as shown in **Figure S3**) is uniquely determined by *n*_prod_, *n*_water_ and *n*_gas_, which are further expressed as functions (*f*, *g* and *h*) of feed glucose concentration (wt%), product yield (g product/g glucose) and/or VVM (min^-1^), as shown in Equations A18-A20. Note that *n*_gas_ is the amount of gas (air and CO_2_) present after fermentation, not the initial total air input through the compressor. MW_prod_ and MW_water_ are the molecular weights of product and water; $\rho_{\mathrm{liq}}=294.75\times glucose concentration+1000$, which is the density of input glucose-water liquid mixture; $\tau$ is residence time; $\sigma$ is gas molar volume; $m_{\mathrm{liq}}$ is the total mass flowrate in the liquid feed; $n_{CO2}$/$n_{O2}$/$\gamma$ is the amount of CO_2_/O_2_/water generated or consumed during fermentation (functions of yield). Note that the glucose mass flowrate in the feed ($m_{\mathrm{glucose}}$) is fixed (40 T/h).

$n_{\mathrm{prod}}=\frac{m_{\mathrm{glucose}}\times\mathrm{yield}}{\mathrm{MW}_{\mathrm{prod}}}=f(yield)$ (A18)

$n_{\mathrm{water}}=\frac{m_{feed water}+\gamma}{\mathrm{MW}_{\mathrm{water}}}=\frac{\frac{100\times m_{\mathrm{glucose}}}{glucose concentration}-m_{\mathrm{glucose}}+\gamma}{\mathrm{MW}_{\mathrm{water}}}=g(glucose concentration, yield)$ (A19)

$n_{\mathrm{gas}}=\frac{m_{\mathrm{liq}}\times\tau\times\mathrm{VVM}}{\sigma\times\rho_{\mathrm{liq}}}+n_{CO2}+n_{O2}=\frac{\left( \frac{100\times m_{\mathrm{glucose}}}{glucose concentration} \right)\times\tau\times\mathrm{VVM}}{\sigma\times\rho_{\mathrm{liq}}}+n_{CO2}+n_{O2}=h(glucose concentration, yield, VVM)$ (A20)

We further express product split fraction to vapor (*α*, i.e., $n_{\mathrm{prod}}^{V}/n_{\mathrm{prod}}$) and liquid product titer (*β*, e.g., g/L) as functions of *n*_prod_, *n*_water_ and *n*_gas_ as shown in Equations A21 and A22. Therefore, they are also functions of glucose concentration, yield and VVM. Implementation of the calculations can be found in Additional File 2.

$\alpha=\frac{n_{\mathrm{prod}}^{V}}{n_{\mathrm{prod}}}=\frac{\min(\frac{f\left( T \right)\times n_{\mathrm{gas}}}{P-f\left( T \right)-g\left( T \right)}, \frac{f\left( T \right)\times\left( n_{\mathrm{gas}}+n_{\mathrm{water}} \right)}{P-f\left( T \right)\text{ }}, n_{\mathrm{prod}})}{n_{\mathrm{prod}}}$ (A21)

$\beta=\frac{m_{\mathrm{prod}}^{L}}{Q^{L}}=\frac{\mathrm{MW}_{\mathrm{prod}}\times(n_{\mathrm{prod}}-\min(\frac{f\left( T \right)\times n_{\mathrm{gas}}}{P-f\left( T \right)-g(T)\text{ }}\text{,} \frac{f\left( T \right)\times\left( n_{\mathrm{gas}}+n_{\mathrm{water}} \right)}{P-f\left( T \right)\text{ }}\text{,}n_{\mathrm{prod}})}{\frac{\mathrm{MW}_{\mathrm{prod}}\times(n_{\mathrm{prod}}-\min(\frac{f\left( T \right)\times n_{\mathrm{gas}}}{P-f\left( T \right)-g(T)\text{ }}\text{,} \frac{f\left( T \right)\times\left( n_{\mathrm{gas}}+n_{\mathrm{water}} \right)}{P-f\left( T \right)\text{ }}\text{,}n_{\mathrm{prod}})}{\rho_{\mathrm{prod}}}+\frac{\mathrm{MW}_{\mathrm{water}}\times(n_{\mathrm{water}}-\min(\frac{g\left( T \right)\times n_{\mathrm{gas}}}{P-f\left( T \right)-g\left( T \right)\text{ }}\text{,}\frac{g\left( T \right)\times\left( n_{\mathrm{gas}}+n_{\mathrm{prod}} \right)}{P-g\left( T \right)\text{ }},n_{\mathrm{water}}\text{)})}{\rho_{\mathrm{water}}}}$ (A22)

**5. VLE analysis in the condenser**

**Figure S4**. VLE analysis applied in the condenser. Parts related to the VLE analysis are marked in green.

Similarly, we apply the VLE analysis in the condenser (see **Figure S4**) by replacing *n*_1_ and *n*_2_ with $n_{\mathrm{prod}}^{V}$ and $n_{\mathrm{water}}^{V}$, and replacing $n_{1}^{V}$, $n_{2}^{V}$, $n_{1}^{L}$ and $n_{2}^{L}$ with $n_{\mathrm{prod}}^{\mathrm{VV}}$, $n_{\mathrm{water}}^{\mathrm{VV}}$, $n_{\mathrm{prod}}^{\mathrm{VL}}$ and $n_{\mathrm{water}}^{\mathrm{VL}}$ (molar flowrates of product and water in the vapor/liquid phase after condensation), respectively. Note that the “min” functions no longer need to be included here because we operate the condenser at temperature *TC* that guarantees co-existence of product and water in the condensed liquid. Thus, we find that $n_{\mathrm{prod}}^{\mathrm{VV}}, n_{\mathrm{water}}^{\mathrm{VV}}$, $n_{\mathrm{prod}}^{\mathrm{VL}} \mathrm{and}n_{\mathrm{water}}^{\mathrm{VL}}$ are functions of $n_{\mathrm{prod}}^{V}, n_{\mathrm{water}}^{V} \mathrm{and} n_{\mathrm{gas}}$, which are functions of glucose concentration, yield and VVM as explained in Section 4. We further express the fraction of product condensed (*λ*) and liquid product concentration after condensation ($\omega$, e.g., g/L) as functions of $n_{\mathrm{prod}}^{V}, n_{\mathrm{water}}^{V} \mathrm{and} n_{\mathrm{gas}}$, as shown in Equations A23 and A24, where *TC* is the condenser temperature. Therefore, *λ* and $\omega$ are also functions of glucose concentration, yield and VVM. Implementation of this calculation can be found in Additional File 2.

$\lambda=\frac{n_{\mathrm{prod}}^{\mathrm{VL}}}{n_{\mathrm{prod}}^{V}}=1-\frac{\frac{f\left( TC \right)\times n_{\mathrm{gas}}}{P-f\left( TC \right)-g(TC)}}{n_{\mathrm{prod}}^{V}}$ (A23)

$\omega=\frac{m_{\mathrm{prod}}^{\mathrm{VL}}}{Q^{\mathrm{VL}}}=\frac{\mathrm{MW}_{\mathrm{prod}}\times(n_{\mathrm{prod}}^{V}-\frac{f\left( TC \right)\times n_{\mathrm{gas}}}{P-f\left( TC \right)-g(TC)})}{\frac{\mathrm{MW}_{\mathrm{prod}}\times(n_{\mathrm{prod}}^{V}-\frac{f\left( TC \right)\times n_{\mathrm{gas}}}{P-f\left( TC \right)-g(TC)})}{\rho_{\mathrm{prod}}}+\frac{\mathrm{MW}_{\mathrm{water}}\times(n_{\mathrm{water}}^{V}-\frac{g\left( TC \right)\times n_{\mathrm{gas}}}{P-f\left( TC \right)-g(TC)\text{ }})}{\rho_{\mathrm{water}}}}$ (A24)

To maximize product recovery, we operate the condenser such that *λ*=100% (all the product is condensed), and the condenser temperature *TC* can be calculated by solving Equation A23 by fixing *λ*=100%, i.e., $f(TC)=0$. Thus, for limonene, *TC* ≅ 0 °C.

**6. Cost distribution by units in Cases 1-3**

**Figure S5**. Cost distribution by units (feedstock costs are excluded; only capital and utility costs are included) for the three cases. (A) Case 1; (B) Case 2; (C) Case 3. Capital costs are annualized.

**Figure S5** shows the cost distribution by units for the three case studies.

**7. Cost distribution by units in Case 4 (use of dodecane solvent)**

**Figure S6**. Cost distribution by units for Case 4 (feedstock costs are excluded; only capital and utility costs are included). Capital costs are annualized.

**Figure S6** shows the cost distribution by units for Case 4. Note that dodecane price is assumed to be 1.5 $/kg. The Antoine constants for dodecane and limonene used to calculate relative volatility for the distillation simulation in SuperPro are as follows, where units of *P* and *T* are mmHg and K, respectively. log_10_*P*=A-B/(*T*+C).

Dodecane: A=6.9979, B=1639.27, C=-91.36

Limonene: A=6.1716, B=1114.54, C=-114.33

**8. Techno-economic parameters**

**Table S1**. Parameters for units.

| **Fermenter** |  | **Compressor** |  | **Condenser** |  | **Centrifuge** |  |
| --- | --- | --- | --- | --- | --- | --- | --- |
| Temperature (C) | 30 | Max outlet temperature (C) | 40 | Pressure (atm) | 1 | Limiting oil globule diameter (μm) | 20 |
| Pressure (atm) | 1 | Pressure change (atm) | 2 | Heat transfer coefficient (W/m2-K) | 860 | Bulk liquid density (g/L) | 1000 |
| Specific power (kW/m3) | 0.3 | Efficiency (%) | 70 | Heat transfer efficiency (%) | 90 | Bulk liquid viscosity (cP) | 1.5 |
| Working to vessel volume ratio (%) | 90 | Labor (labor hrs/hr) | 0.05 | Temperature difference factor | 1 | Efficiency (%) | 30 |
| Labor (labor hrs/hr) | 1 |  |  | Labor (labor hrs/hr) | 0 | Product removal (%) | 100 |
|  |  |  |  |  |  | Cells removal (%) | 95 |
|  |  |  |  |  |  | Power dissipation to heat (50%) | 50 |
|  |  |  |  |  |  | Labor (labor hrs/hr) | 1 |

**Table S2**. Parameters for capital costs.

|  | **Fermenter** | **Compressor** | **Condenser** | **Centrifuge** |
| --- | --- | --- | --- | --- |
| PC (purhcase cost) Base size | 3500 m3 | 2982 KW | 95 m2 | 27 m3/h |
| PC Base cost ($) | 700000 | 3236000 | 39000 | 258000 |
| PC power law exponent | 0.6 | 0.6 | 0.6 | 0.6 |
| Max size per equipment | 2750 m3 | 3000 KW | 100 m2 | 9000 m3/h |
| Installation cost (% of PC) | 30 | 50 | 50 | 50 |
| Annual maintenance cost (% of PC) | 10 | 10 | 10 | 10 |

**Table S3**. Parameters for feedstock and utility costs, and other parameters.

| **Parameter** | **value** |
| --- | --- |
| Glucose ($/kg) | 0.6 |
| Water ($/m3) | 0.85 |
| Dodecane ($/kg) | 1.5 |
| Chilled water ($/T) | 0.4 |
| Cooling water ($/T) | 0.05 |
| Freon ($/Mcal) | 0.1 |
| Electricity ($/KW-h) | 0.1 |
| Costing year | 2016 |
| Operating days per year | 330 |
| Operating capacity (%) | 100 |
| Capital charge factor (calculated) | 0.188 |

**9. SuperPro screenshot**


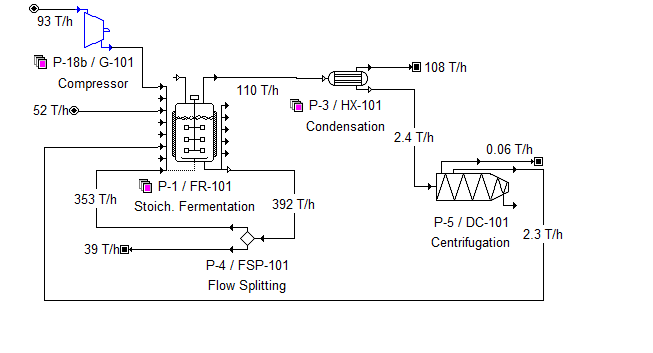

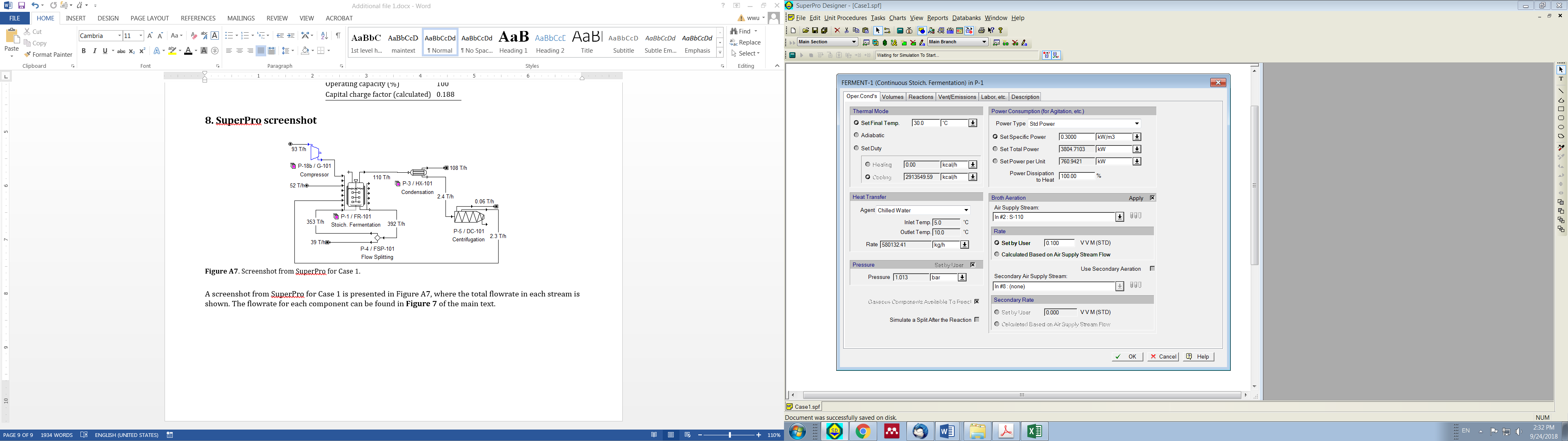


**Figure S7**. Screenshot from SuperPro for Case 1, including the process flowsheet and the interface for fermentation modeling as an example.

A screenshot from SuperPro for Case 1 is presented in **Figure S7**, where the total flowrate in each stream is shown. The flowrate for each component can be found in **Figure 7** of the main text.
